# Apoplexy, Paralysis, Softening of the Brain—A Plea for Non-Performance of Promise of Marriage

**Published:** 1856-03

**Authors:** W. B. Kesteven


### *Apoplexy, Paralysis, Softening of the Brain—A Plea for Non-Performance of Promise of Marriage.*

[To the Editor of the Medical Times and Gazette.]

The Dublin newspapers of December 15th, kindly forwarded to me by Mr. Wilde, report a trial for breach of promise of marriage, presenting features of much interest in a Medico-legal point of view.

The morbid conditions alleged in defence have been generally recorded in Medico-legal works, so far as I have been able to discover, as a cause of impotence, and have not been brought forward in a court of justice as the ground of defence in an action similar to the present. I have, therefore, thought that the present case might be acceptable to your readers.

#### Hurford *v.* Singleton.

This action was defended on the ground that at the time defendant had promised marriage he was advanced in life, viz., 60 years of age; and that before a reasonable time had elapsed from the request to marry, namely, in May, 1855, he was, by a visitation of God, attacked by a fit of apoplexy, since which time he was in an infirm state, and afflicted with softening of the brain, in consequence of which he could not perform his promise without putting his life into great peril, and hastening his death.

Evidence was called on the part of the plaintiff to prove the engagement, and to show that apparent impairment of health or vigor remained after recovery from the attack.

It was stated by the defendant's counsel, Mr. Ball, that, in 1849, he had suffered from dropsy and disease of the kidneys; that, in 1852, he had an attack of apoplexy and congestion of the brain. During the interval from that time until May last, he had promised to marry the plaintiff; but that in the latter month he was afflicted with another attack of apoplexy, and was now suffering from paralysis and softening of the brain.

The Medical evidence in support of the defence, was as follows :—

Dr. Speedy had attended the defendant, with Mr. Cusack, in 1849, when suffering under dropsy, and again in 1852, when he had the attack of apoplexy. Dr. Speedy stated that he is now liable to another attack, and is paralytic ;—further, that an attack of apoplexy would endanger his life. Upon being asked whether marriage would put the life of defendant in peril, Dr. Speedy replied, that the circumstances under which another attack might be produced would depend upon his physical powers. The witness would not give it as his opinion that he might not consummate the marriage without periling his life ; he had known many instances of delicate persons marrying, and getting stronger and better afterwards.

Mr. Wilde had known defendant fourteen or fifteen years ; he had seen him in June last, when he found him laboring under paralysis ; the defendant had a similar attack in 1852 ; also a serious varicose ulcer on his leg from 1851 to 1854, off and on ; he is now partially paralyzed in the leg and arm, and suffering from loss of memory. Mr. Wilde had doubts whether he could consummate matrimony. As one of the Census Commissioners, Mr. Wilde knew that defendant was not competent to fulfil the duties of the appointment he held in the Census Office. On cross-examination, Mr. Wilde stated that he had known men get married after apoplexy ; he had not known an instance of a person with an arm or a leg paralyzed getting married. He agreed with Dr. Speedy's opinion.

Mr. Cusack had attended defendant in 1852 for an apoplectic attack ; also in May last, when he had an apoplectic attack, and was becoming paralytic. Mr. Cusack believed that any excitement would increase the tendency to another attack, and would tend to hasten his death.

The jury returned a verdict for the plaintiff, £300 damages, and costs.

The ground of this verdict, it is said, was that the jury considered that an unreasonable time had elapsed between the date of the promise of marriage and the date of the last attack of apoplexy.

" The sexual function," observes Dr. Taylor, " is so intimately allied to bodily vigor and nervous energy, that the integrity of the one may be pronounced to be essential to the other," ("Medical Jurisprudence," 5th edit. page 625.) Relatively to the case before us, we may add, that the one will surely react upon the other. It is well known that, not only does excessive sexual indulgence induce paralysis, but that even moderate, and only occasional, sexual intercourse has been followed by fatal consequences, in persons of delicate health. The occurrence of hemorrhage from internal organs, the lungs, brain, etc., has frequently followed upon copulation; still more probable was it, that it should take place in a brain damaged by softening—a pathological condition of the brain very commonly ending in either apoplexy or paralysis.

The defence, therefore, set up in this case was a valid defence. The general evidence did not show other than a disposition to perform the promise of marriage previously to the attack, although the jury, it appears, thought the period that had elapsed was unreasonably long. However this may be, there can be little room to doubt that the opinions expressed by Dr. Speedy, Mr. Wilde and Mr. Cusack were strictly correct, and established the defence so far as the plea rested upon the state of the defendant's health. That such heavy damages should have been awarded on account of the lapse of time is matter of considerable surprise.

I am, etc., W. B. Kesteven.

